# Finding the Beat: From Socially Coordinated Vocalizations in Songbirds to Rhythmic Entrainment in Humans

**DOI:** 10.3389/fnhum.2016.00255

**Published:** 2016-06-06

**Authors:** Jonathan I. Benichov, Eitan Globerson, Ofer Tchernichovski

**Affiliations:** ^1^Department of Psychology, Hunter College, City University of New YorkNew York, NY, USA; ^2^Gonda Multidisciplinary Brain Research Center, Bar-Ilan UniversityRamat-Gan, Israel; ^3^Jerusalem Academy of Music and DanceJerusalem, Israel

**Keywords:** songbird vocalizations, zebra finch, social coordination, rhythm, vocal learning, predictive timing, entrainment, rhythm perception

## Abstract

Humans and oscine songbirds share the rare capacity for vocal learning. Songbirds have the ability to acquire songs and calls of various rhythms through imitation. In several species, birds can even coordinate the timing of their vocalizations with other individuals in duets that are synchronized with millisecond-accuracy. It is not known, however, if songbirds can perceive rhythms holistically nor if they are capable of spontaneous entrainment to complex rhythms, in a manner similar to humans. Here we review emerging evidence from studies of rhythm generation and vocal coordination across songbirds and humans. In particular, recently developed experimental methods have revealed neural mechanisms underlying the temporal structure of song and have allowed us to test birds' abilities to predict the timing of rhythmic social signals. Surprisingly, zebra finches can readily learn to anticipate the calls of a “vocal robot” partner and alter the timing of their answers to avoid jamming, even in reference to complex rhythmic patterns. This capacity resembles, to some extent, human predictive motor response to an external beat. In songbirds, this is driven, at least in part, by the forebrain song system, which controls song timing and is essential for vocal learning. Building upon previous evidence for spontaneous entrainment in human and non-human vocal learners, we propose a comparative framework for future studies aimed at identifying shared mechanism of rhythm production and perception across songbirds and humans.

Almost all animals behave in reference to physical and biological rhythms. From the entrainment of a cricket's circadian cycles, to a sandpiper's repeated chasing and retreating from the waves on a shoreline, rhythms, and synchronization are ubiquitous in animal behavior (Strogatz, 2003). Animals do not only adapt to rhythms, but they can also generate coordinated rhythmic patterns, as in the synchronous flashing of fireflies or the antiphonal calling of marmosets (Moiseff and Copeland, 1995; Takahashi et al., 2013). Although rhythms, entrainment, and coordination appear to be widespread, some highly intelligent animals, such as dogs and apes, appear limited in their ability to spontaneously synchronize their actions to a given beat (Merker, 2000; Fitch, 2011), whereas most humans can dance and can synchronize their movements to a broad range of beats with ease. What is it that makes entrainment so easy for a few animal species (Large and Gray, 2015; Wilson and Cook, 2016) including humans, and difficult or impossible for others?

Many animals communicate by exchanging rhythmic calls. Such rhythms might simply arrise from stereotyped back and forth responses to individual stimuli (Figure 1A). Alternatively animals might respond to sequences of events (Figure 1B) or to temporal pattens (Figure 1C), that is, to the overall periodicity of events. In the case of sequences, one may ask if the animal has learned and responded to a simple or a complex string of contiguous events (Figure 1B). In the case of rhythm learning, the experimental question is whether the animal is capable of anticipating the timing of events, which may be either equally spaced in time (i.e., isochrounous events, Figure 1C, top) or complex(i.e., hierarchically organized events corresponding to a musical meter, Figure 1C, bottom).

**Figure 1:**
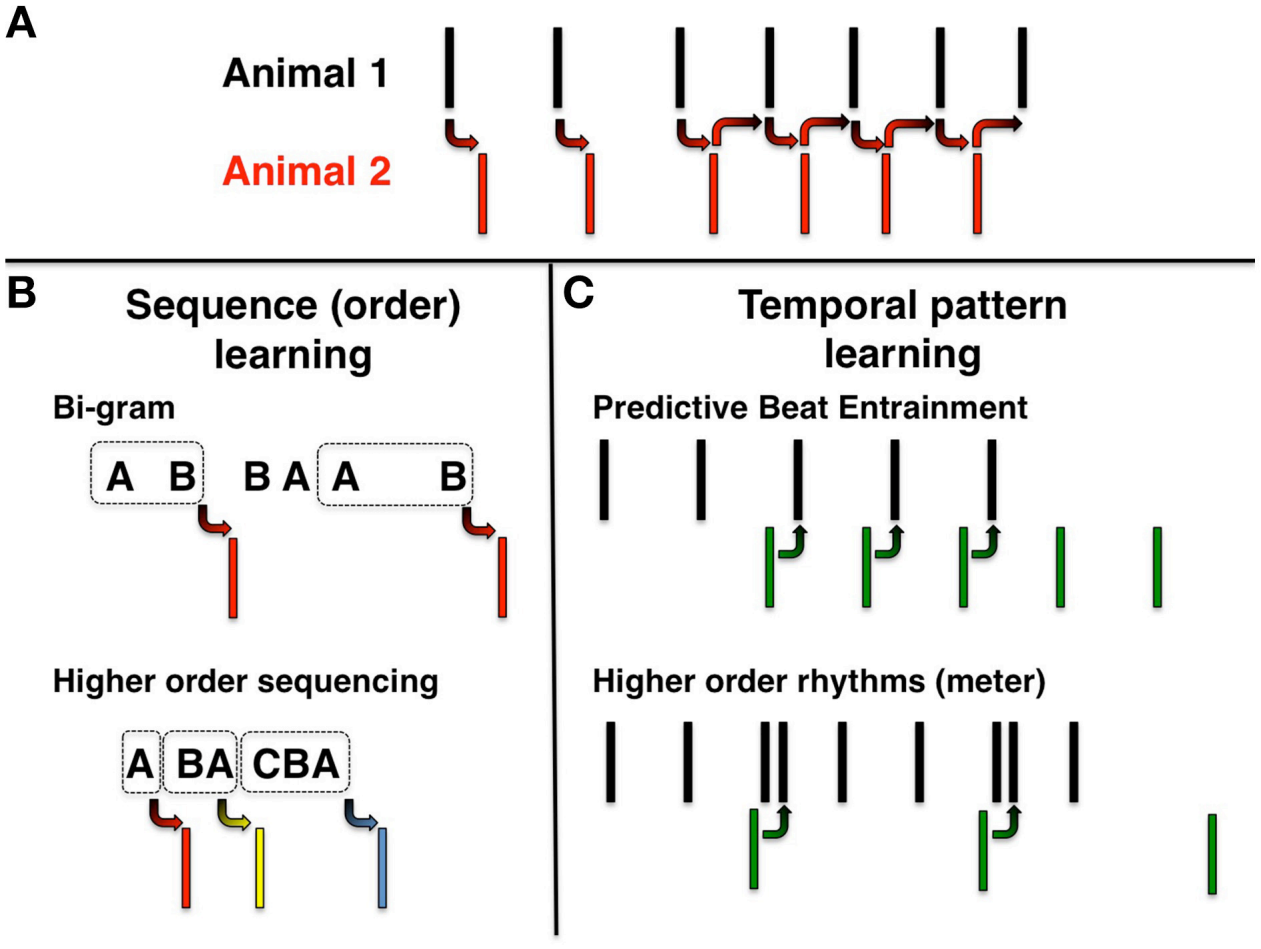
**Rhythmic behavior. (A)** Simple stimulus-response behavior. Left: Animal 2 respond to calls of Animal 1. Right: A stimulus-response loop between two animals generating sustained oscillations. **(B)** Sequence learning. Top: Animal responds to pairwise call sequences (bi-grams). Bottom: Responses to higher order sequential patterns. **(C)** Rhythm learning. Top-left: Green predictive calls (negative asynchrony) to external beat with constant period (isochronous pattern). Top-right: Calls continue with the same beat after stimulus has stopped (entrainment). Bottom: Negative asynchrony and entrainment to a meter.

Here we review emerging evidence from studies of rhythm generation and vocal coordination across songbirds and humans. We start with a brief review of rhythm learning assessment in human subjects, and present the difficulties in using comparable approaches in animal studies of rhythm learning. We then discuss recent approaches to studying how songbirds can coordinate their vocalization in reference to an external beat.

## Characteristics of rhythm entrainment in humans

In humans, contrary to many other species, rhythmic entrainment is a universal feature of behavior, observed from a very early age (Fraisse, 1966). Typically, when human subjects are asked to synchronize their movement to a beat, they tend to anticipate it. That is, they tend to act just prior to the onset of the beat, an effect called *negative asynchrony* (Figure 1C, top). Negative asynchrony can be observed after just a few (2–3) introductory beats and typically precedes the stimulus by tens of milliseconds (Fraisse, 1966). This effect can be observed over a fairly broad range of inter-stimulus intervals (ISIs), between 100 and 1800 ms. The lower ISI bound is determined by motor constraints, while the higher bound is believed to be our limited ability to detect periodicity beyond certain tempi (e.g., due to memory and attention constraints). Above the 100–1800 ms range, negative asynchrony partly gives way to positive asynchrony, which reflects responsive, rather than predictive synchronization to the beat (Fraisse, 1982). Another typical human trait in beat perception is the tendency to create a hierarchy of beats, commonly termed *meter* (Figure 1C, bottom). Grouping of beats is also typical of human motor entrainment to a beat. When moving to music, humans tend to perceive levels of hierarchy beyond the basic beat and synchronize their movement to these groups of beats (Palmer and Krumhansl, 1990).

## Evaluating rhythm learning in non-human animals

When reviewing animal studies of rhythm learning we will broadly consider cases where animals can learn to adjust their behavior with respect to a given rhythm. The choice of behavioral indicators of rhythmic perception, however, is non-trivial. The basic supposition underlying behavioral paradigms is that perception drives behavior, and, in some cases, is modulated by behavior. In human studies, one can affect behavior by directly instructing subjects to respond to perception with certain informative actions. A non-human animal, however, will respond to a stimulus only if it corresponds to a meaningful event according to some species-typical standards. Lack of response can reflect perceptual or behavioral limitations, but also the lack of motivation to respond. Therefore, in testing animal rhythm perception, it is critical to find auditory stimuli that are salient enough for the animal to respond in an informative manner. In cases where it is possible to elicit reliable responses, studies typically focus on responses as individual events, rather than ongoing patterns.

Entrainment to rhythms can be tested in many presumably reflexive responses which may exhibit the signatures of rhythm perception or recognition of a beat (Figure 1C). With behaviors that appear to be periodic, this can be tested simply by shifting the phase of a repeated stimulus or by observing the persistence of the periodic behavior after the removal of the entraining rhythmic stimuli (Figure 1C). Such manipulations are, in fact, standard when studying circadian rhythms in animals (Panda, 2002), but rare in animal communication studies. Singing behavior in oscine songbirds is one of the most studied systems of communication. Birdsong is learned, complex, and often highly rhythmic. Remarkably, some songbird species can even coordinate their songs during duets in which they alternate song syllables with millisecond accuracy (Yoshida and Okanoya, 2005; Fortune et al., 2011; Templeton et al., 2013; Rivera-Cáceres, 2015). Inspired by this rich behavior, several recent studies have involved manipulation of rhythmic stimuli to examine rhythm perception and entrainment in songbirds (Lampen et al., 2014; van der Aa et al., 2015; Benichov et al., 2016).

## Vocal learning and coordination in songbirds

There are about 4000 species of oscine songbirds, many of which produce songs, which are learned, culturally transmitted behaviors (Brenowitz and Beecher, 2005). Songs are extremely diverse in their spectro-temporal features, complexity, and usage across species (Beecher and Brenowitz, 2005). A European nightingale male, for example, typically learns hundreds of different songs, and sings them in an enormously complex succession. A male zebra finch, on the other extreme, typically learns only a single song motif during development. Within those species-specific constraints, each individual bird can be recognized by its unique song, which is often partly learned through imitation, and partially improvised. Interestingly, even the songs of birds raised in complete isolation possess individual rhythm signatures (Fehér et al., 2009).

### The forebrain song system is a generator of complex learned rhythms

The neuronal mechanisms of song learning and production have been studied in great detail (Brainard and Doupe, 2002; Nottebohm, 2005). It appears that song patterns (Amador et al., 2013) originate in a highly localized brain center, nucleus HVC (used as proper name), which is located in the bird's posterior forebrain (Nottebohm, 2005). Premotor HVC neurons, which project downstream to primary motor centers, are active during singing, and their spikes are extremely sparse and accurate (Kozhevnikov and Fee, 2007). For example, the zebra finch song is composed of a repeated sequence e.g., *ABCD, ABCD*…where each letter represents a syllable type and the repeated unit [*ABCD*] is called a motif. Each premotor HVC neuron produces only a single short burst of action potentials during a motif, “ticking” at a specific “moment” (e.g., in the middle of syllable C). Collectively, the ticks of these neurons span the entire duration of the song motif and cooling HVC while the bird sings, results in the slowing of the song, with an almost perfectly uniform reduction in tempo across its duration (Long and Fee, 2008). This result suggests that nucleus HVC is the principle generator of song structure. In a recent study (Okubo et al., 2015), the activity of HVC neurons was tracked during developmental song learning. Interestingly, during early development, HVC neurons generate much faster rhythms, often time-locked to a single prototype syllable, which the bird produces in rapid succession (Tchernichovski et al., 2001; Aronov et al., 2008). The prototype syllable then gradually differentiates into several mature syllable types. For example, a chain of prototype syllables *XXXX* may transform into *XX*′*XX*′, and finally into *ABAB*. As this differentiation takes place, HVC neurons double their ticking period, such that they gradually shift from bursting once per syllable, to bursting once every other syllable (on either A or B), until eventually, HVC neurons spike only once per song motif (e.g., *ABCD*…).

Is the emergence of rhythmic patterns simply mirroring the process of learning to imitate sequences of syllable types? Alternatively, are rhythms the primary skeleton of song production and perception? We do not know. The song system could be either a sequence generator that appears to be rhythmic or a developing rhythm generator, where HVC neurons are, in effect, entrained by the auditory memory of perceived rhythms. If the latter is correct, then through the capacity of song learning, songbirds are endowed with neuronal mechanisms that are specialized for acquiring rhythms. Interestingly, nuclei in the zebra finch auditory association cortex, which are known to be involved in song recognition, are highly sensitive to rhythmic song patterns (Lampen et al., 2014). The authors presented birds with modified songs, where the sequential order of song elements remained unchanged, but song rhythm was perturbed by randomly varying inter-syllable intervals (arrhythmic songs). Hearing arrhythmic songs strongly increased activity in auditory brain areas compared to rhythmically natural songs, supporting the notion that rhythmic structure is a salient feature of birdsong, both for males and non-singing females.

Songbirds are, perhaps, a rare example of animals in which predictive auditory-motor synchronization has evolved. If song learning is indeed a neural entrainment of HVC to memories of perceived rhythms, duet singing could be interpreted as real-time coupling of song rhythms between two birds. For example, in the plain tailed wren, both females and males sing. They learn and perform impressive duets, alternating song syllables in perfect synchrony as if one bird were singing (Rivera-Cáceres, 2015). Notably, premotor HVC neurons are sensitive to the intervals of the dueting partner (Fortune et al., 2011). In sum, the vocal learning capacities of songbirds enable them to create highly complex song patterns, and employ them in social communication, but we don't know if these patterns are primarily perceived and produced as sequences (song syntax), as rhythms (temporal structures), or perhaps as both.

There are few conclusive studies of entrainment to rhythms in non-human animals, most famously in the form of “dancing” in parrots (Patel et al., 2009; Schachner et al., 2009; Hasegawa et al., 2011; Laland et al., 2016). As noted earlier, birdsong is often highly rhythmic, and the song system can be thought of as a sophisticated generator of learned rhythmic behavior. However, once learned, song rhythms become highly stereotyped and difficult to manipulate. In contrast, it is much easier to assess rhythmic vocal abilities when birds are exchanging calls. Zebra finches, for example, rapidly exchange innate short calls and coordinate the timing of their short calls in a pair-specific manner while in a group of calling birds (Elie et al., 2010; Anisimov et al., 2014; Ter Maat et al., 2014). Recently, the vocal coordination capacity of zebra finches has been tested under controlled conditions, in terms that potentially allow for direct comparison to human rhythmic entrainment studies. These experiments showed that zebra finches can coordinate the timing of simple unlearned calls with an imposed beat in a manner that is predictive (Benichov et al., 2016).

**Figure 2:**
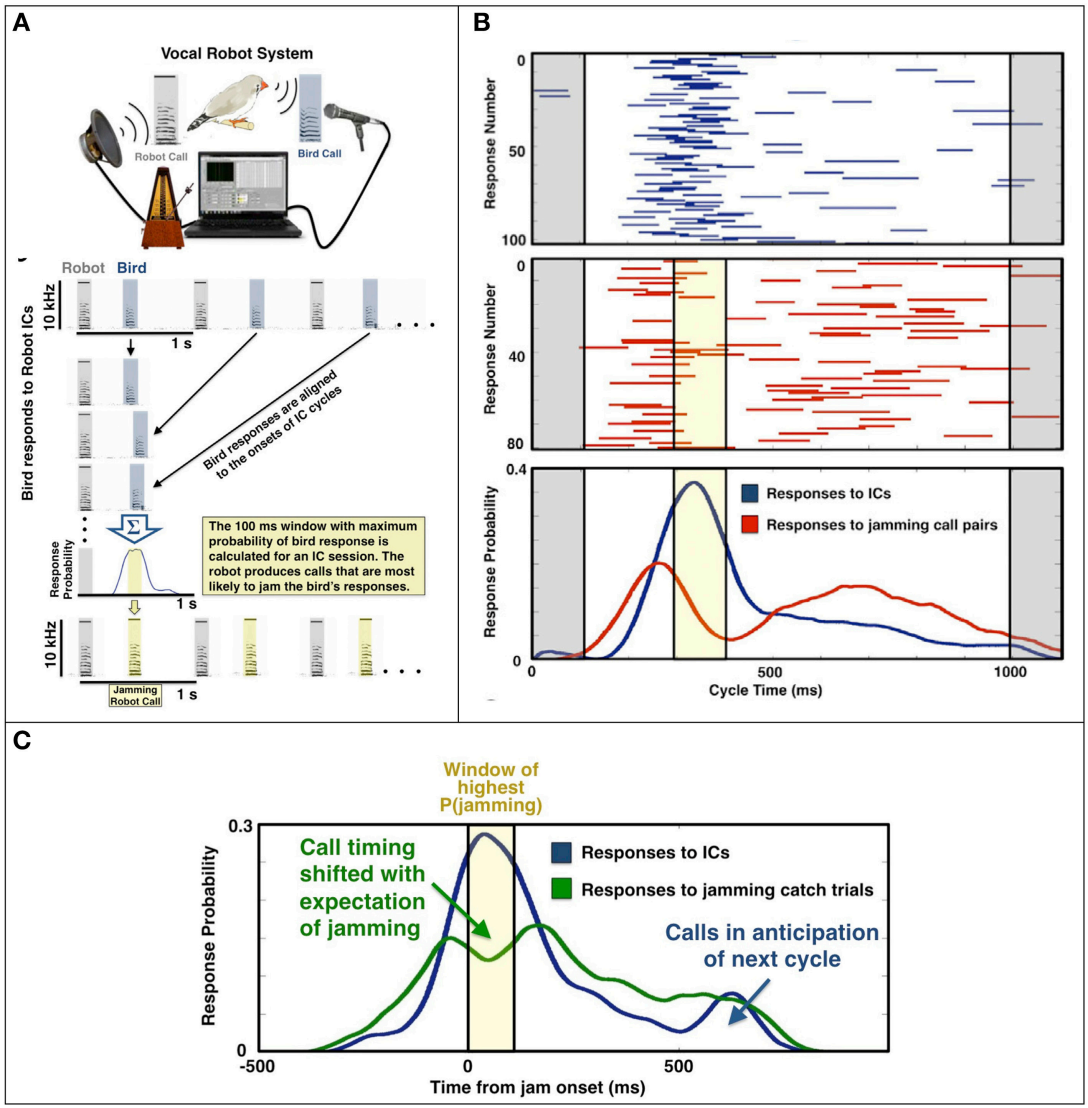
**Vocal Robot and jamming avoidance (From Benichov et al., 2016). (A)** A bird interacts with isochronous calls (ICs) generated by a vocal robot at rate of 1 Hz. The bird's stereotyped response latencies are used to determine a window of maximum jamming probability (yellow). **(B)** A bird's responses (blue) across 1000 ms robot IC cycles (gray) and responses (red) across a subsequent session containing jamming robot calls (yellow). The bird shifts its response probability distribution to avoid jamming. **(C)** Cumulative response distributions across 12 birds, aligned to their window of maximum jamming probability (yellow), for ICs (blue), and for jamming catch trials (green) that contain only a single robot call.

In an initial task, individual birds were presented with equally spaced (isochronous) calls (ICs) from a vocal robot (Figure 2A) and answered the robot calls with stereotyped latencies. The robot then generated pairs of calls, with intervals that matched the bird's typical response time, thereby maximizing the likelihood of jamming (Figure 2A, bottom). Within seconds, birds learned to alter the timing of their responses to avoid jamming (Figure 2B). We showed that timing adjustments were predictive, that is, birds anticipated the jamming and shifted timing accordingly (Figure 2C). This was verified with “catch” trials, or occasional cycles in which birds hear a single call within a session consisting primarily of jamming call pairs. Further, like humans anticipating a beat, birds typically adjusted their call timing after hearing only a few cycles of the pattern.

Exchanges of calls with the vocal robot typically take the form of antiphonal duets, as opposed to in-phase synchrony. Jamming avoidance can then be thought of as a mechanism for maintaining antiphony. In comparison to human beat perception, call anticipation underlying jamming avoidance may be analogous to the expectation of a beat that underlies temporal shifts in syncopated rhythms (Fitch and Rosenfeld, 2007; Velasco and Large, 2011; Nozaradan et al., 2016). In both cases, events do not occur on the beat, but rather, they are shifted relative to the expectation of the beat. In music this is employed and perceived as accenting, whereas in zebra finches, this anticipation appears to guide antiphonal coordination. These results make sense from an ecological perspective given that zebra finches typically exchange thousands of short calls daily, and their colonies tend to be dense and busy acoustic environments (Elie et al., 2011).

## A comparative approach for studying behavioral mechanisms of rhythm learning

Jamming avoidance has been thoroughly studied in several species of weakly-electric fishes (Bullock et al., 1972; Heiligenberg et al., 1996; Zupanc and Bullock, 2006) and frogs (Zelick and Narins, 1982, 1985). These animals minimize signal overlap with their neighbors by adjusting their intrinsic pacemaker intervals, cycle-by-cycle (Zelick, 1986). Generalized phase resetting and shifting mechanisms constitute responsive forms of coordination. For example, phase adjustment mechanisms can explain how the coqui frog can avoid jamming by preferably calling during brief periods of silence (Zelick and Narins, 1985). Are these animals learning to synchronize their signals (i.e., to cooperate)? Can synchrony arise as an epiphenomenon of competitive interactions (i.e., by suppression)? In the case of chorusing Kaydid bush crickets, females prefer the “leader” male that starts to signal just prior to his competitors, in a manner that resembles negative asynchrony (Greenfield and Roizen, 1993; Fertschai et al., 2007; Hartbauer et al., 2014). In this case, sexual selection for competitive inhibitory mechanisms, which are primarily responsive, may account for the apparent synchrony of the chorus, without the need for prediction.

In zebra finches, vocal robot experiments have shown that birds predictively adjust the timing of their calls when presented with complex rhythms (Benichov et al., 2016). Beyond shifting call timing for repeated jamming call pairs, zebra finches also make anticipatory adjustments for alternating jamming and non-jamming cycles. Birds appear to predict the pattern and reduce response latencies specifically for cycles in which jamming calls occur. These non-generalized (i.e., context-sensitive) shifts in response latencies cannot be explained by responsive mechanisms alone. Rather, they would require mechanisms that can operate on longer time scales (e.g., sequences) or multiple temporally hierarchical levels (e.g., grouping of beats). To our knowledge, such context-sensitive plasticity has not been observed in signaling insects, electric fish, or frogs.

Despite the impressive context-sensitive plasticity in songbird call timing, there is no conclusive evidence that they can perceive rhythms holistically, as humans do. The human ability to perceive rhythms and exhibit spontaneous sensorimotor entrainment has certain hallmarks: it is predictive, occurs across multiple hierarchical timescales, and exhibits predictive negative asynchrony enabled by endogenous representation of an isochronous beat (Semjen et al., 1998; Merker et al., 2009; Nozaradan et al., 2016). It also occurs within a specific range of tempi (Fraisse, 1982). These features can provide a starting point for comparative studies. Along these lines, van der Aa et al. tested rhythm perception in songbirds, and found that zebra finches could not generalize a distinction between isochronous and irregular beats across tempi, namely, they failed to categorize rhythms based on their common global temporal patterns (van der Aa et al., 2015). Humans, in contrast, can easily perform such tasks without any prior training regardless of cultural background (Merker et al., 2009). These results suggest that songbirds attend to local timing events in a sequence but not to global rhythm patterns.

A “sequence-based” explanation could potentially account for predicative call timing plasticity (Benichov et al., 2016). In this scenario birds detect local contiguities of events and adjust their call timing according to a rule of succession. For example, a bird might learn to answer more quickly after hearing a long interval and more slowly after hearing a short interval (Figure 1B). Even though zebra finches attend to local acoustic patterns during passive listening, it remains possible that they can synchronize their calls to a given beat in the context of vocal interactions. Indeed, preliminary evidence may suggest a rhythm entrainment mechanism: when zebra finches interact with a vocal robot, a surprising proportion of calls occur just before the next anticipated robot call, as in negative asynchrony or anticipatory “leading” (Figure 2C, secondary peak in IC responses). To further test if call timing reflects predictive entrainment to the previously heard robot rhythm, we are currently analyzing persistent call patterns produced by birds after a robot call pattern has been terminated (Figure 1C).

## A comparative approach for studying brain mechanisms of rhythm learning

MEG and EEG studies in humans have shown neural entrainment to an external beat (Honing et al., 2014; Doelling and Poeppel, 2015; Nozaradan et al., 2016). No similar phenomenon has been reported in non-human animals that exhibit spontaneous rhythm synchronization or in songbirds. The lack of evidence in songbirds studies might mirror technical difficulties: As discussed earlier, the forebrain song system is a highly specialized vocal learning network. However, song learning proceeds over weeks, and is difficult to manipulate from moment to moment. The vocal robot approach for studying rhythm adaptation, and possibly entrainment of calls, could facilitate such comparative experiments.

Recent studies have identified zebra finch brain areas that drive call timing and interestingly, the forebrain song system appears to play a major role. The first evidence for song system involvement came from electrophysiological studies, showing the final premotor output nucleus, RA (robust nucleus of the archopallium), is active during the exchange of unlearned short calls (Ter Maat et al., 2014). These findings were surprising given that birds can exchange such calls even after the output of the forebrain song system has been blocked (Simpson and Vicario, 1990; Aronov et al., 2008). However, performing jamming avoidance experiments while the song system is lesioned or blocked results in complete loss of a bird's ability to synchronize its calls with a robot partner (Benichov et al., 2016). While birds remain responsive to the robot calls, their latencies become significantly less stereotyped. This was accompanied by the dramatic loss of the ability to avoid jamming. The precise timing of call coordination, therefore, relies on forebrain circuits that also underlie song learning.

What could this mean? Interestingly, female zebra finches, who do not sing, are extremely good at avoiding jamming. In fact, their jamming avoidance behavior is more accurate than that of male zebra finches (Benichov et al., 2016). Could the female “song system” be involved in vocal coordination? The female zebra finch forebrain vocal nuclei are not well developed (Nottebohm and Arnold, 1976; Wade and Arnold, 2004), yet blocking nucleus RA disrupted call timing and jamming avoidance, as it did in males. Therefore, the female song system, which was assumed to be vestigial, functions in call coordination and is perhaps more highly specialized for the task than the male's forebrain vocal pathway. Together, findings suggest that the song system involvement in the coordination of unlearned calls reflects the interplay between sensory prediction and motor control. Consequently, blocking the cortical output of the song production pathway results in the temporal uncoupling of the birds' calls from the robot's, as measured by a loss of response precision and predictive timing adjustments. This occurred without affecting the birds' tendency to respond to robot calls. As the search for the neural mechanisms of call coordination narrows down, it should be possible to test if single neurons can be entrained to an imposed beat in songbirds, and to compare the results directly to human studies of neuronal entrainment.

In comparison to humans, the behavioral results obtained after blocking the song system may in some ways be analogous to the rhythm deficits seen in some human subjects who have difficulty synchronizing to an external beat (Amos, 2013) or have been identified as “beat deaf” (Phillips-Silver et al., 2011). Understanding the roles of sensorimotor networks underlying temporal deficits in songbirds may provide insights for related human research. At this point, it is too early to judge the extent to which the control of adaptive call timing is localized to the song system. Other brain areas, particularly, the descending auditory pathway (Mello et al., 1998), which surrounds the song system, is likely to be involved as well. This would be consistent with reports of top-down modulation of auditory processing in human subjects (Tervaniemi et al., 2009). However, since the song system has the capacity to generate and perhaps entrain to song rhythms, an extension of this capacity to call timing adjustments would be a reasonable explanation for the anatomical convergence of the two. In sum, it should now be possible to test if neuronal activity in any of the forebrain song nuclei can be entrained to rhythms produced by a vocal robot in behaving birds. If successful, such experiments should allow for direct comparisons to human rhythm learning experiments, both at neuronal and behavioral levels.

## Author contributions

All authors listed, have made substantial, direct and intellectual contribution to the work, and approved it for publication.

## Funding

This work was funded by grants to OT from the National Institute on Deafness and Other Communication Disorders (National Institutes of Health: 5R01DC004722-17), the National Science Foundation (IOS-1261872), and the Professional Staff Congress of the City University of New York (66810-00 44).

### Conflict of interest statement

The authors declare that the research was conducted in the absence of any commercial or financial relationships that could be construed as a potential conflict of interest.
